# Prevalence and Distribution of Apical Periodontitis in Root Canal-Treated Teeth: A Cone-Beam Computed Tomography Study in a Saudi Subpopulation

**DOI:** 10.3390/diagnostics16040618

**Published:** 2026-02-20

**Authors:** Obadah Austah, Lama Alghamdi, Amjad Alshamrani, Taggreed Wazzan, Mohammed Barayan, Mohammed A. Alharbi, Abdullah Bokhary, Loai Alsofi

**Affiliations:** 1 Department of Endodontics, Faculty of Dentistry, King Abdulaziz University, P.O. Box 80209, Jeddah 21589, Saudi Arabia; lamaalghamdi997@gmail.com (L.A.); dr.amjadalshamrani@gmail.com (A.A.); maealharbi@kau.edu.sa (M.A.A.); 2 Division of Oral and Maxillofacial Radiology, Department of Oral Diagnostic Sciences, University Dental Hospital, King Abdulaziz University, P.O. Box 80209, Jeddah 21589, Saudi Arabia; twazzan@kau.edu.sa; 3 Division of Oral and Maxillofacial Radiology, Department of Oral Diagnostic Sciences, Faculty of Dentistry, King Abdulaziz University, P.O. Box 80209, Jeddah 21589, Saudi Arabia; mbarayan@kau.edu.sa; 4 Department of Dental Public Health, Faculty of Dentistry, King Abdulaziz University, P.O. Box 80209, Jeddah 21589, Saudi Arabia; amabokhary@kau.edu.sa

**Keywords:** apical periodontitis, cone-beam computed tomography, CBCT, root canal treatment, AP-RCT, prevalence, endodontics, epidemiology, periapical disease

## Abstract

**Background:** Apical periodontitis (AP) is a common inflammatory condition of the periapical tissues, most often associated with persistent endodontic infection. Conventional two-dimensional radiography may underestimate AP because of anatomical superimposition and limited sensitivity. Cone-beam computed tomography (CBCT) allows three-dimensional visualization of periapical structures and has been increasingly used in epidemiological research. **Objective:** This study aimed to evaluate the prevalence and distribution of apical periodontitis, with particular emphasis on apical periodontitis associated with root canal-treated teeth (AP-RCT), in a Saudi subpopulation using CBCT imaging. **Methods:** This retrospective cross-sectional study analyzed CBCT scans of Saudi patients obtained for routine diagnostic purposes between 2017 and 2021. Apical periodontitis was identified using standardized radiographic criteria requiring the presence of periapical radiolucency in more than one imaging plane. Demographic and clinical variables were recorded. Descriptive statistics were used to estimate prevalence. Associations between demographic factors and AP-RCT counts were evaluated using multivariable negative binomial regression. Regional tooth distribution was analyzed using generalized estimating equation models accounting for within-participant clustering. **Results:** A total of 320 CBCT scans were analyzed. Apical periodontitis was detected in 231 participants (72.2%) and in 667 teeth (8.3% of examined teeth). Of the affected teeth, 457 (68.5%) were associated with root canal treatment. The mean number of AP-RCT per participant was 1.36 ± 1.81 (median: 1; IQR: 0–2). Multivariable analysis identified age as the only significant predictor of AP-RCT. Compared with individuals aged 21–30 years, higher AP-RCT rates were observed in the 31–40-year and 41–50-year age groups, while participants ≤20 years showed lower rates. Tooth-level analysis demonstrated higher AP-RCT prevalence in maxillary premolars, maxillary molars, and mandibular molars, whereas mandibular anterior teeth showed the lowest prevalence. **Conclusions:** Apical periodontitis, particularly AP-RCT, was frequently observed in this Saudi subpopulation when assessed using CBCT. Age and tooth location were the primary determinants of disease distribution. These findings provide population-level epidemiological data on the prevalence and anatomical distribution of apical periodontitis in root canal-treated teeth. Clinical Significance: CBCT-based epidemiological assessment enables detailed evaluation of the distribution of apical periodontitis in dentate populations and may assist in characterizing disease patterns in anatomically complex regions, without implying comparative diagnostic accuracy or treatment outcome assessment.

## 1. Introduction

Apical periodontitis (AP) is an inflammatory condition of the periapical tissues caused primarily by microorganisms and other byproducts within the root canal system [1]. Microbial infection is therefore the principal etiological factor in disease development and a major cause of persistent or recurrent AP following root canal treatment, particularly when the technical quality of the endodontic therapy or coronal restoration is compromised [2]. Clinically, AP may be asymptomatic, and although pain, sensitivity, or swelling can occur, clinical signs and symptoms alone are insufficient for establishing a definitive diagnosis [3]. Consequently, diagnosis relies on the combined interpretation of clinical findings, diagnostic tests, and radiographic assessment. While histological examination remains the gold standard for identifying apical periodontitis [4,5], it is not feasible in routine clinical practice, rendering radiographic evaluation essential for diagnosis and treatment planning.

Conventional radiographic techniques, including two-dimensional (2D) periapical and panoramic radiographs, have historically been used to assess periapical status. However, these modalities provide a 2D representation of a three-dimensional (3D) structure and lack the buccolingual dimension, which limits their diagnostic accuracy. Cone-beam computed tomography (CBCT) has gained widespread adoption in endodontics due to its ability to eliminate anatomical superimposition and provide high-resolution three-dimensional visualization of periapical structures [6,7,8]. Detection of apical periodontitis using conventional 2D imaging is particularly challenging, as periapical radiolucency typically becomes evident only when cortical bone involvement occurs, with lesions confined to cancellous bone frequently remaining undetected. Experimental studies have shown that approximately 30–50% mineralized bone loss is often required before periapical lesions become radiographically visible on 2D images [9,10]. In endodontic practice, CBCT has demonstrated superior diagnostic performance for identifying apical pathology, evaluating complex canal anatomy, detecting root fractures and resorptive defects, and supporting surgical treatment planning [8,11].

The prevalence of apical periodontitis has been investigated in several populations using different imaging modalities. Consistently, studies have reported a higher prevalence of AP in root canal-treated teeth, with disease occurrence strongly influenced by the quality of endodontic treatment and coronal restorations. A recent systematic review and meta-analysis reported a global prevalence of approximately 52% at the individual level and 5% at the tooth level, highlighting the substantial burden of AP worldwide [4,12].

In Saudi Arabia, several studies have evaluated the prevalence of AP using panoramic or periapical radiographs [12,13,14,15]. However, some investigations included mixed-nationality cohorts or focused exclusively on endodontically treated teeth, limiting the generalizability of their findings [12,13,14]. More recently, CBCT-based assessment has been employed; however, available studies were restricted to posterior root canal-treated teeth only [4]. Given the inherent limitations of 2D imaging, reliance on conventional radiography may underestimate the true prevalence and distribution of AP in the Saudi population. To date, a comprehensive CBCT-based epidemiological assessment of apical periodontitis involving all permanent teeth in Saudi individuals remains lacking.

Therefore, the aim of this study was to estimate the prevalence and anatomical distribution of apical periodontitis detected on CBCT in a Saudi subpopulation, with emphasis on apical periodontitis in root canal-treated teeth (AP-RCT). Secondary objectives were to evaluate demographic and clinical predictors of AP-RCT and to describe region- and tooth-level distribution patterns.

## 2. Materials and Methods

### 2.1. Sample Selection

Ethical approval for this retrospective cross-sectional study was obtained from the Research Ethics Committee, Faculty of Dentistry, King Abdulaziz University (Approval No. 311-11-21). CBCT scans of Saudi patients were retrieved from the Radiology Department database at King Abdulaziz University Dental Hospital and were retrospectively collected for the period between January 2017 and January 2021.

Data retrieval, anonymization, and eligibility screening were completed in 2021 following ethical approval. All CBCT scans were originally acquired for routine clinical diagnostic purposes, including implant planning, evaluation of maxillofacial pathoses, and general dental assessment; no scans were obtained specifically for research purposes or selected based on the presence or absence of apical pathology. All records were fully anonymized prior to analysis.

**Figure 1:**
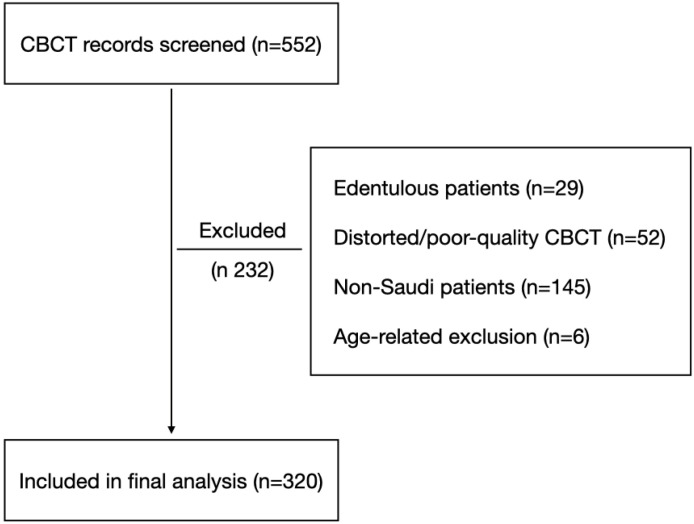
Flowchart illustrating the selection of CBCT records included in the study. A total of 552 CBCT records were screened. Records were excluded due to edentulism, poor-quality or distorted CBCT images, non-Saudi nationality, or age-related criteria. The final sample consisted of 320 CBCT records included in the analysis.

Inclusion criteria comprised Saudi nationality; dentate patients with fully erupted permanent teeth; medium or large field-of-view (FOV) CBCT scans capturing both dental arches; scans of acceptable diagnostic quality without significant distortion or artifacts; and a minimum age of 12 years. CBCT records were excluded if patients were edentulous, if scans were distorted or of poor diagnostic quality, if patients were non-Saudi, or if they fell outside the predefined age range. The CBCT scan selection process is illustrated in Figure 1. All eligible medium/large-FOV CBCT scans meeting inclusion criteria during the study period were included consecutively from the institutional database.

### 2.2. Radiographic Evaluation

All CBCT scans were acquired using an iCAT CBCT scanner (Imaging Sciences International, Hatfield, PA, USA) with a 16 × 10 cm field of view, operating at 120 kVp and 5–7 mA, and reconstructed with a voxel size of 0.2 mm. Images were evaluated using Vision software (version 1.6.20) (Imaging Sciences International, Hatfield, PA, USA) on a calibrated 17-inch high-resolution monitor in a dimly lit, windowless room to optimize image interpretation. Contrast and magnification settings were adjusted as required.

For each CBCT scan, data were collected at the tooth level using a standardized recording form, documenting the presence or absence of apical periodontitis, root canal treatment status, tooth type, and anatomical location. All assessments were performed systematically for each tooth within the field of view to ensure consistent and complete data extraction.

**Figure 2:**
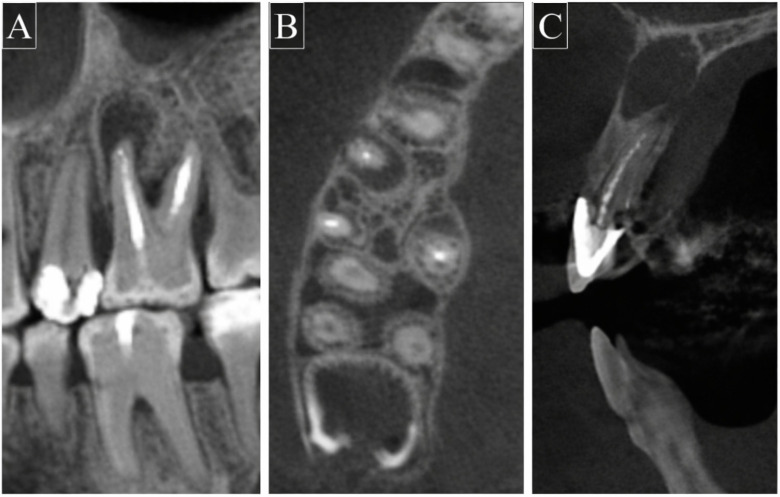
Representative cone-beam computed tomography (CBCT) images demonstrating apical periodontitis in root canal-treated teeth. (**A**) Coronal view showing periapical radiolucency associated with the treated root of tooth #26. (**B**) Axial view illustrating periapical radiolucency at the root apex of tooth #16. (**C**) Sagittal view confirming the periapical radiolucency associated with tooth #11.

Each CBCT volume was assessed in the axial, sagittal, and coronal planes. A diagnosis of apical periodontitis was recorded only when a periapical radiolucency was visible in more than one imaging plane. Apical periodontitis was defined according to the criteria described by De Moor et al. [16] as a periapical radiolucency associated with the apical portion of the root exceeding twice the normal width of the periodontal ligament space. Representative CBCT images illustrating the diagnostic criteria are shown in Figure 2.

CBCT data collection followed a standardized and predefined protocol. All scans were retrieved retrospectively from the institutional radiology database, anonymized prior to evaluation, and assessed using consistent imaging parameters and software settings. Each scan was systematically reviewed in axial, sagittal, and coronal planes according to predefined diagnostic criteria for apical periodontitis. Examiner calibration and reproducibility procedures were conducted before data extraction to ensure consistency and methodological reliability.

Demographic and health-related variables, including age, sex, and general medical history, were recorded. Additional variables included the total number of teeth present, tooth type, tooth location (sextant), and the presence or absence of root canal treatment.

### 2.3. Examiner Calibration

Three examiners independently performed the CBCT assessments. Prior to data collection, all examiners underwent standardized training and calibration using representative CBCT cases and a predefined scoring protocol. Intra-examiner reproducibility was assessed by re-evaluating 20 randomly selected teeth after a two-week washout period, demonstrating almost perfect agreement (Cohen’s κ = 0.89). Inter-examiner agreement was evaluated on the same calibration set and indicated substantial to almost perfect agreement across examiner pairs (κ = 0.66–0.88). Any disagreements during the main evaluation were resolved by consensus, with verification by a senior oral and maxillofacial radiologist when necessary.

### 2.4. Sample Size Calculation

This retrospective cross-sectional study included all eligible CBCT scans available during the predefined study period (2017–2021). Based on previously reported CBCT-based prevalence estimates of apical periodontitis (approximately 5–10%), a sample size of approximately 300 participants was considered sufficient to estimate prevalence with a 95% confidence level and acceptable precision. The final sample of 320 participants, therefore, provided adequate statistical power for prevalence estimation and multivariable regression analyses.

### 2.5. Statistical Analysis

All statistical analyses were conducted using SAS OnDemand for Academics (SAS Studio; SAS 9.4; SAS Institute Inc., Cary, NC, USA). Descriptive statistics were used to summarize demographic and clinical characteristics. Categorical variables are presented as frequencies and percentages, while continuous and count variables are summarized as mean ± standard deviation (SD) and median (interquartile range, IQR).

The primary inferential analysis focused on apical periodontitis associated with root canal-treated teeth (AP-RCT), defined as the number of teeth with apical periodontitis and prior root canal-treated teeth per participant. Given the over dispersed count nature of this outcome, associations with predictors were evaluated using negative binomial regression with a log link. The multivariable model included age category, sex, smoking status, ASA classification, medical history (dichotomized as none/insignificant versus any medical condition), and total number of teeth present. Results are reported as adjusted incidence rate ratios with 95% confidence intervals (CI) and two-sided *p*-values.

To assess the regional distribution of AP while accounting for within-participant correlation, region-level analyses were performed by restructuring the data to long format with six regions per participant (maxillary anterior, premolar, molar, mandibular anterior, premolar, molar). Regional differences were evaluated using generalized estimating equation (GEE) negative binomial regression, incorporating an offset for the log number of teeth present in each region and adjusting for the same covariates used in the primary model.

For tooth-level analyses, the presence or absence of apical periodontitis was modeled as a binary outcome per tooth, separately for AP-RCT and AP-virgin, using GEE logistic regression to account for clustering of teeth within participants. Models were adjusted for age category, sex, smoking status, ASA classification, and medical history. The mandibular central incisor (tooth 31) was selected a priori as the reference category due to its low baseline risk for apical periodontitis. Third molars were excluded. Statistical significance was set at *p* < 0.05 (two-sided).

## 3. Results

### 3.1. Participant Characteristics

The demographic and clinical characteristics of the 320 participants are summarized in Table 1. The cohort was predominantly medically healthy, with most participants classified as ASA I (80.0%), while 16.6% and 3.4% were classified as ASA II and ASA III, respectively. The age distribution was weighted toward younger and middle-aged adults, with individuals aged 21–30 years representing the largest group (31.3%), followed by those aged 31–40 years (24.7%) and 41–50 years (17.5%). Smaller proportions were observed at the extremes of age, with 6.9% aged ≤ 20 years and 10.6% aged ≥ 61 years. Females comprised 56.9% of the study population. Most participants reported no or insignificant medical history (79.4%) and were non-smokers (94.1%).

### 3.2. Dentition Characteristics and Apical Periodontitis Burden

Participants-level dentition characteristics and apical periodontitis burden are presented in Table 2. The study population retained a substantial dentition, with a mean of 24.88 ± 5.42 teeth per participant (median: 26; IQR: 24–28). Apical periodontitis was detected in an average of 2.02 ± 2.27 teeth per participant (median: 1; IQR: 0–3). Apical periodontitis associated with root canal-treated teeth (AP-RCT) constituted the majority of disease burden (mean 1.36 ± 1.81; median: 1; IQR: 0–2), whereas apical periodontitis in non-root canal-treated teeth (AP-virgin) was less frequent (mean 0.66 ± 1.25; median: 0; IQR: 0–1).

### 3.3. Factors Associated with Apical Periodontitis in Root Canal-Treated Teeth

Results of the multivariable negative binomial regression examining factors associated with AP-RCT burden are shown in Table 3. After adjustment for demographic and clinical covariates, age emerged as the primary determinant of AP-RCT burden. Compared with participants aged 21–30 years, significantly higher AP-RCT counts were observed among those aged 31–40 years (IRR = 2.00, 95% CI: 1.39–2.86; *p* < 0.001) and 41–50 years (IRR = 1.91, 95% CI: 1.27–2.88; *p* = 0.002). Participants aged ≤ 20 years demonstrated a significantly lower AP-RCT burden (IRR = 0.45, 95% CI: 0.20–0.99; *p* = 0.047). No statistically significant associations were observed for sex, smoking status, ASA classification, medical history, or total number of teeth present. The lack of association with dentition extent indicates that differences in AP-RCT burden were not explained by variations in the number of remaining teeth (IRR = 0.99, 95% CI: 0.97–1.02; *p* = 0.699).

### 3.4. Regional Distribution of Apical Periodontitis

The region-based generalized estimating equation (GEE) negative binomial model evaluating apical periodontitis rates per tooth present is presented in Table 4. Compared with the upper anterior region, significantly higher AP rates were observed in the upper premolar (IRR = 1.36; 95% CI: 1.04–1.78; *p* = 0.025), upper molar (IRR = 1.57; 95% CI: 1.18–2.09; *p* = 0.002), and lower molar regions (IRR = 1.48; 95% CI: 1.11–1.96; *p* = 0.006). In contrast, the lower anterior region demonstrated a substantially lower AP rate (IRR = 0.29; 95% CI: 0.19–0.46; *p* < 0.001). The lower premolar region did not differ significantly from the reference (IRR = 0.77, 95% CI: 0.55–1.09; *p* = 0.145).

Age remained independently associated with regional AP rates. Compared with participants aged 21–30 years, higher rates were observed in the 31–40, 41–50, 51–60, and ≥61 years age groups, whereas participants aged ≤ 20 years did not differ significantly from the reference (IRR = 1.82; 95% CI: 1.30–2.52; *p* < 0.001, IRR = 1.73; 95% CI: 1.16–2.57; *p* = 0.007, IRR = 1.83; 95% CI: 1.11–3.01; *p* = 0.018, IRR = 2.04; 95% CI: 1.36–3.08; *p* < 0.001, IRR = 0.63; 95% CI: 0.37–1.09; *p* = 0.099, respectively). No significant associations were observed for sex (*p* = 0.635), smoking status (*p* = 0.150), ASA class (ASA II: *p* = 0.159; ASA III: *p* = 0.150), or medical history (*p* = 0.229) in this adjusted model.

### 3.5. Tooth-Level Distribution of Apical Periodontitis

Tooth-level analyses demonstrated marked heterogeneity in the likelihood of apical periodontitis by tooth position (Table 5A,B). For apical periodontitis in root canal-treated teeth (AP-RCT), significantly higher odds were predominately observed in posterior teeth, particularly maxillary and mandibular molars, compared with the mandibular central incisor reference. The strongest associations were observed for maxillary and mandibular first molars, with adjusted odds ratios approaching tenfold increases in AP-RCT likelihood (OR 9.84; 95% CI: 3.50–27.62; *p* < 0.001, OR 9.52; 95% CI: 3.39–26.74; *p* < 0.001, respectively). Several premolars also demonstrated significantly elevated odds, whereas anterior teeth generally showed no significant differences from the reference (e.g., tooth 25, *p* = 0.055; tooth 44, *p* = 0.057, teeth 32, 33, and 41–43; all *p* > 0.40).

For apical periodontitis in non-root canal-treated teeth (AP-virgin), tooth-level differences were less pronounced, and estimates were generally less precise, reflecting the lower frequency of disease in untreated teeth. Statistically significant associations were primarily observed in posterior teeth, particularly mandibular molars (OR 4.50; 95% CI: 1.25–16.17; *p* = 0.021), while most anterior and premolar teeth did not differ significantly from the reference.

## 4. Discussion

This retrospective cross-sectional study evaluated the prevalence and distribution of apical periodontitis in a Saudi subpopulation using CBCT. Consistent with previous investigations, the findings demonstrate a high burden of apical periodontitis, particularly in root canal-treated teeth, and highlight the diagnostic advantages of CBCT over conventional 2-dimensional radiographic modalities.

Multiple studies have shown that AP of endodontic origin may remain undetected on conventional radiographs, particularly when lesions are confined to cancellous bone or masked by anatomic superimposition. Comparative investigations consistently report higher detection rates of apical lesions when CBCT is used, reflecting its superior sensitivity and 3-dimensional visualization. These findings support the use of CBCT as a more accurate diagnostic tool for assessing periapical status, particularly in epidemiological research where underestimation of disease prevalence is a recognized limitation of 2-dimensional imaging [9,10,17].

CBCT provides three-dimensional visualization with higher diagnostic accuracy than conventional radiography and has been recommended as an adjunct imaging modality when conventional radiographs may not adequately depict periapical pathology, provided that its use is clinically justified and radiation exposure is optimized [18].

Velvart et al. correlated radiographic findings from conventional imaging and computed tomography with surgical observations of periapical lesions and their relationship to adjacent anatomical structures. They demonstrated that all lesions identified during surgery were visible on CT scans, whereas only 78.2% were detected using periapical radiographs [17]. Similarly, Jang et al. reported a 10.8% probability of missing apical periodontitis when using periapical radiographs [19]. Lofthag-Hansen et al. identified 53 lesions visible on both 2D and 3D images, in addition to 33 lesions detected exclusively on CBCT scans [10]. Collectively, these findings support the superior sensitivity and diagnostic accuracy of CBCT for detecting apical lesions compared with conventional radiographic modalities.

All CBCT scans included in the present study were acquired for routine diagnostic purposes unrelated to apical pathology alone, thereby reducing the likelihood of targeted selection based on the presence of periapical disease. Nevertheless, the study population cannot be considered fully representative of the general population, as CBCT imaging is typically reserved for complex diagnostic or treatment-planning scenarios rather than routine screening. This reliance on clinically indicated CBCT datasets may therefore introduce a degree of selection bias, potentially overrepresenting patients with more complex dental conditions. Despite this limitation, the higher prevalence of apical periodontitis observed in the present study compared with reports from other populations is likely attributable, at least in part, to the enhanced diagnostic sensitivity of three-dimensional CBCT imaging compared with conventional radiographic methods.

Several criteria have been proposed in the literature for the diagnosis of AP. The periapical index (PAI), developed by Ørstavik et al. (1986) [20], provides a five-point scoring system originally designed for periapical radiographs and has been widely applied in studies using periapical and panoramic imaging. In the present study, AP was defined according to the criteria proposed by De Moor et al. (2000) [16], in which a periapical radiolucency surpassing twice the width of the normal periodontal ligament space is considered diagnostic. This definition has been widely adopted in CBCT-based epidemiological studies and allows for a more reliable and standardized assessment of periapical pathology using 3-dimensional imaging.

Apical periodontitis was detected in 72% of participants and 8.3% of teeth, values that exceed tooth-level prevalence estimates reported in several CBCT-based studies from European and South American populations, where prevalence ranged from approximately 3% to 6% [3,4,12,13,14]. Differences in sampling strategies, diagnostic criteria, examiner calibration, and population oral health profiles may contribute to these variations [21]. Importantly, the present findings further support the notion that conventional radiography may underestimate the true prevalence of apical periodontitis, particularly when compared with 3-dimensional imaging. Estrela et al. demonstrated that CBCT detects significantly more apical lesions than periapical and panoramic radiographs, with markedly higher sensitivity, particularly for early or less advanced lesions that are frequently missed on conventional imaging [22]. This diagnostic limitation of two-dimensional radiography likely contributes to the lower prevalence figures reported in studies relying solely on periapical or panoramic radiographs.

In Saudi Arabia, most previously published prevalence studies relied on panoramic or periapical radiographs and reported lower prevalence estimates [13,15]. For instance, Alnazhan et al. examined teeth from panoramic radiographs and reported a prevalence of apical periodontitis of 6.2%. The higher prevalence observed in this CBCT-based study reinforces concerns regarding the limitations of 2-dimensional imaging in detecting periapical pathology and suggests that the burden of apical periodontitis in the Saudi population may be greater than previously reported.

A substantial proportion of apical lesions in the present study (68.5%) were associated with root canal-treated teeth, consistent with findings from both regional and international studies. This association likely reflects a combination of factors, including inadequate cleaning and shaping, suboptimal obturation, and deficiencies in coronal restoration integrity, all of which may permit bacterial persistence or re-infection of the root canal system. In addition, the absence of vital pulp tissue and the biofilm-driven nature of both AP and periodontal disease may further contribute to disease persistence in treated [23].

The relatively high prevalence of AP in root canal-treated teeth observed in the present study exceeds that reported in many studies using conventional radiography (typically 6–12%) [15,24,25,26] and is slightly higher than figures reported in several CBCT-based investigations (approximately 50–60%) [27,28]. This discrepancy may reflect the higher sensitivity of CBCT for detecting small, residual, or healing periapical lesions that may not be apparent on two-dimensional images. Recent CBCT-based studies have similarly reported elevated AP-RCT prevalence and emphasized that three-dimensional imaging frequently identifies periapical changes reflecting healing, scar tissue, or bone remodeling rather than active inflammatory disease. In contrast to the present findings, Hussain et al. reported a substantially lower prevalence of apical periodontitis in root canal-treated teeth (31.7%) in an adult Nepalese subpopulation using panoramic radiographs, highlighting the influence of imaging modality and diagnostic sensitivity on reported prevalence estimates [29].

Importantly, the presence of apical periodontitis in root canal-treated teeth should not be interpreted as definitive evidence of endodontic treatment failure. Detection of AP-RCT is influenced by multiple factors, including obturation length and density, coronal restoration quality, host response, and the high sensitivity of CBCT, which may reveal persistent or healing periapical changes not visible on conventional radiographs. Consequently, AP-RCT identified on CBCT does not necessarily indicate active disease or unsuccessful treatment but may reflect residual or remodeling lesions detected due to advanced imaging resolution [30]. Recent CBCT studies have further demonstrated that technically adequate root canal treatments may still present with radiographically detectable periapical changes, particularly in the form of residual or remodeling lesions, underscoring that AP-RCT identified on CBCT does not necessarily indicate persistent infection or treatment failure [28]. Consistent with this interpretation, Nascimento et al. demonstrated that apical periodontitis was significantly associated with endodontic technical errors and inadequate coronal restorations on CBCT, highlighting that radiographically detected AP in root-filled teeth is strongly influenced by treatment quality rather than treatment failure alone [31].

Central incisors were therefore selected as the reference category in tooth-level regression analyses, as they represent teeth with the lowest baseline risk for apical periodontitis. This is attributable to their relatively simple root canal anatomy, lower caries incidence, and more favorable access for endodontic procedures. Using a low-risk reference group allows clearer interpretation of relative odds across other tooth types and emphasizes the disproportionate disease burden observed in posterior teeth.

Tooth- and region-level analyses demonstrated marked heterogeneity in AP distribution. Posterior teeth, particularly maxillary and mandibular molars, exhibited the highest disease burden, whereas mandibular anterior teeth consistently showed the lowest prevalence. The increased susceptibility of posterior teeth may be attributed to their complex root canal anatomy, earlier eruption, prolonged functional exposure, and higher caries risk, all of which complicate effective endodontic treatment. In contrast, the lower prevalence observed in mandibular anterior teeth is consistent with their lower caries susceptibility and simpler canal morphology [32,33].

Regarding tooth location, maxillary teeth exhibited a higher prevalence of AP (63%) compared with mandibular teeth (37%), consistent with previous studies [3]. Maxillary molars and maxillary anterior teeth demonstrated the highest prevalence (23% each), findings that agree with earlier reports [3,12,13,14].

Age emerged as the only independent predictor of AP associated with root canal-treated teeth. Higher disease burden was observed in middle-aged and older individuals, whereas younger participants demonstrated significantly lower prevalence. This trend likely reflects cumulative exposure to caries, restorations, and endodontic interventions over time, as well as age-related changes in oral health status [34]. Consistent with the present findings, Hussain et al. identified the quality of root canal treatment as the strongest predictor of apical periodontitis, with inadequately treated teeth exhibiting an approximately eight-fold increased risk of AP. In addition, the authors reported a protective effect of female sex and of permanent coronal restorations, while age and tooth type were not independent predictors in multivariable analysis [29]. Previous CBCT-based analyses have shown that technical factors such as underfilling, missed canals, and poor coronal restorations are significant predictors of apical periodontitis in root canal-treated teeth, underscoring the importance of incorporating standardized treatment quality assessments in future epidemiological studies [31]. Age may therefore act as a surrogate for cumulative dental interventions and longer post-treatment observation periods rather than indicating age-related biological susceptibility alone.

Apical periodontitis was most prevalent among patients aged 30–39 years and least prevalent among those under 20 years of age, supporting previous reports that prevalence increases with age [12,13,14]. Although a higher crude prevalence was observed among females, sex was not independently associated with AP after multivariable adjustment, suggesting that this difference may reflect population characteristics rather than biological susceptibility [34].

Most previous studies have reported no significant sex-related differences in the prevalence of AP [3,14,15]. In contrast, the present study found a higher prevalence among females (58%) compared with males (42%). However, sex was not independently associated with AP-RCT in adjusted regression analyses, suggesting that this difference may reflect distributional rather than biological factors. The reasons for this difference remain unclear and warrant further investigation. Recent CBCT-based evidence has highlighted that sex-related differences in apical periodontitis may be more closely related to lesion characteristics than prevalence alone. Estrela et al. reported a higher overall prevalence of apical periodontitis in females, whereas males demonstrated a greater proportion of larger and more severe osteolytic lesions on CBCT [35]. These findings suggest a potential sexual dimorphism in disease expression, with differences in severity and progression rather than simple occurrence.

Several limitations should be considered when interpreting these findings. The retrospective design precluded assessment of important clinical variables such as the technical quality of root canal treatment and the integrity of coronal restorations. In addition, although CBCT provides high-resolution three-dimensional imaging and superior sensitivity compared with conventional radiography, the diagnosis of apical periodontitis in this study was based exclusively on radiographic criteria rather than histological confirmation. Consequently, CBCT findings cannot differentiate with absolute certainty between active inflammatory apical periodontitis, healing lesions, fibrous scar tissue, or other non-inflammatory periapical bone changes. As a cross-sectional investigation, the study captures disease status at a single time point and therefore does not permit evaluation of lesion progression or resolution. Although CBCT offers superior spatial resolution, its use must be justified on clinical grounds and interpreted within the constraints of radiographic diagnosis, as emphasized by professional guidelines that caution against indiscriminate use and highlight the inability of CBCT to provide histological confirmation [18]. Furthermore, ethical and radiation-related considerations limit the feasibility of population-based CBCT screening, necessitating reliance on scans acquired for other diagnostic indications, which may introduce selection bias. Despite these limitations, the present study provides valuable insight into the epidemiology of apical periodontitis using high-resolution three-dimensional imaging.

Furthermore, the absence of detailed treatment quality parameters, including obturation length, density, homogeneity, and coronal restoration integrity, represents an important limitation. These factors are well-established determinants of apical periodontitis in root canal-treated teeth and may modify or confound the observed associations, particularly those related to age and anatomical distribution. Incorporation of standardized assessments of endodontic and restorative quality in future CBCT-based studies would allow more precise differentiation between technical, biological, and patient-related contributors to AP-RCT. Inclusion of treatment quality variables may attenuate the observed association between age and AP-RCT if older age primarily reflects greater exposure to root canal treatment and restorative history.

Despite these limitations, the present study provides robust population-level data on the distribution of apical periodontitis using CBCT and highlights the strengths and constraints of radiographic-based epidemiological assessment.

## 5. Conclusions

Within the limitations of this retrospective cross-sectional study, apical periodontitis was frequently detected in this Saudi subpopulation, with the majority of affected teeth being root canal-treated. Apical periodontitis associated with root canal-treated teeth (AP-RCT) was significantly associated with increasing age and demonstrated marked anatomical variation, with a higher disease burden in posterior teeth, particularly molars. In contrast, sex, smoking status, ASA classification, and general medical history were not independently associated with AP-RCT after multivariable adjustment.

These findings provide population-level epidemiological data on the prevalence and distribution of apical periodontitis in a Saudi subpopulation assessed using CBCT and should be interpreted within the context of a radiographic-based cross-sectional design.

### Clinical Significance

CBCT-based epidemiological assessment enables detailed evaluation of the anatomical distribution of apical periodontitis in dentate populations. These data may assist clinicians and health systems in identifying higher-burden tooth regions and planning targeted preventive and monitoring strategies, without implying treatment outcome assessment.

## Figures and Tables

**Table 1:** Demographic and Clinical Characteristics of the Study Participants (n = 320).

Characteristic	Category	n (%)
ASA status	ASA I	256 (80.0)
	ASA II	53 (16.6)
	ASA III	11 (3.4)
Age (years)	≤20	22 (6.9)
	21–30	100 (31.3)
	31–40	79 (24.7)
	41–50	56 (17.5)
	51–60	29 (9.1)
	≥61	34 (10.6)
Gender	Female	182 (56.9)
	Male	138 (43.1)
Medical history	No/insignificant	254 (79.4)
	Any medical condition	66 (20.6)
Smoking status	No	301 (94.1)
	Yes	19 (5.9)

Data are presented as numbers (n) and percentages (%); ASA = American Society of Anesthesiologists physical status; smoking reflects current smoking status.

**Table 2:** Participant-Level Dentition Characteristics and Apical Periodontitis Burden (n = 320).

Variable	Mean ± SD	Median
Teeth per individual	24.88 ± 5.42	26.00 (24.00–28.00)
Apical Periodontitis (AP)	2.02 ± 2.27	1.00 (0.00–3.00)
AP-RCT	1.36 ± 1.81	1.00 (0.00–2.00)
AP-virgin	0.66 ± 1.25	0.00 (0.00–1.00)

Values represent participant-level counts summarized across all participants (n = 320) and are presented as mean ± SD and median.

**Table 3:** Multivariable Negative Binomial Regression for Apical Periodontitis in Root Canal-Treated Teeth: Adjusted Incidence Rate Ratios (n = 320).

Predictor	Category	Adjusted IRR (95% CI)	*p*-Value
ASA status	ASA I	1.00 Ref	-
	ASA II	0.68 (0.18–2.52)	0.562
	ASA III	0.52 (0.11–2.37)	0.396
Age (years)	21–30	1.00 Ref	-
	≤20	0.45 (0.20–0.99)	0.047
	31–40	2.00 (1.39–2.86)	<0.001
	41–50	1.91 (1.27–2.88)	0.002
	51–60	1.53 (0.88–2.65)	0.132
	≥61	0.98 (0.54–1.75)	0.934
Gender	Female	1.00 Ref	-
	Male	0.87 (0.66–1.15)	0.322
Medical history	No/insignificant	1.00 Ref	-
	Any medical condition	1.40 (0.39–5.07)	0.609
Smoking status	No	1.00 Ref	-
	Yes	1.41 (0.83–2.41)	0.205
Total number of teeth	Per additional tooth	0.99 (0.97–1.02)	0.699

Adjusted incidence rate ratios were estimated using negative binomial regression and are presented with 95% confidence intervals (CI). Reference categories were ASA I, age 21–30 years, female, no/insignificant medical history, and non-smoker. “Total number of teeth” represents the effect per additional tooth present. *p*-value < 0.05 was considered statistically significant.

**Table 4:** Region-Based GEE Negative Binomial Model for Apical Periodontitis Rate per Tooth Present: Adjusted Incidence Rate Ratios (n = 320).

Predictor	Category	Adjusted IRR (95% CI)	*p*-Value
Region	Upper anterior	1.00 Ref	-
	Upper premolar	1.36 (1.04–1.78)	0.025
	Upper molar	1.57 (1.18–2.09)	0.002
	Lower anterior	0.29 (0.19–0.46)	<0.001
	Lower premolar	0.77 (0.55–1.09)	0.145
	Lower molar	1.48 (1.11–1.96)	0.006
ASA status	ASA I	1.00 Ref	-
	ASA II	0.57 (0.26–1.25)	0.159
	ASA III	0.54 (0.23–1.25)	0.150
Age (years)	21–30	1.00 Ref	-
	≤20	0.63 (0.37–1.09)	0.099
	31–40	1.82 (1.30–2.52)	<0.001
	41–50	1.73 (1.16–2.57)	0.007
	51–60	1.83 (1.11–3.01)	0.018
	≥61	2.04 (1.36–3.08)	<0.001
Gender	Female	1.00 Ref	-
	Male	1.06 (0.83–1.36)	0.635
Medical history	No/insignificant	1.00 Ref	-
	Any medical condition	1.58 (0.75–3.31)	0.229
Smoking status	No	1.00 Ref	-
	Yes	1.55 (0.85–2.83)	0.150

Adjusted incidence rate ratios were estimated using a GEE negative binomial regression. Reference categories were upper anterior region, ASA I, age 21–30 years, female, no/insignificant medical history, and non-smoker. *p*-value < 0.05 was considered statistically significant.

**Table 5:** Tooth-Level Association with Apical Periodontitis (GEE Logistic Regression). (**A**) Tooth-Level Association with Apical Periodontitis in Root Canal-Treated Teeth: Adjusted Odds Ratios. (**B**) Tooth-Level Association with Apical Periodontitis in Non-Root Canal-Treated Teeth: Adjusted Odds Ratios.

Tooth	Adjusted OR (95% CI)	*p*-Value	Tooth	Adjusted OR (95% CI)	*p*-Value
(**A**)					
11	8.27 (2.83–24.12)	<0.001	31	1.00 Ref	-
12	4.46 (1.46–13.59)	0.009	32	1.05 (0.37–2.95)	0.931
13	2.55 (0.78–8.34)	0.120	33	1.13 (0.42–3.01)	0.803
14	5.59 (1.87–16.72)	0.002	34	3.75 (1.40–10.06)	0.009
15	3.36 (1.07–10.56)	0.038	35	2.55 (0.93–7.00)	0.071
16	9.84 (3.50–27.62)	<0.001	36	5.59 (1.96–15.95)	0.001
17	3.44 (1.10–10.74)	0.033	37	3.75 (1.35–10.40)	0.011
21	5.88 (2.06–16.75)	0.001	41	1.05 (0.37–2.95)	0.931
22	4.74 (1.56–14.36)	0.006	42	1.49 (0.57–3.90)	0.416
23	3.36 (1.19–9.53)	0.022	43	1.30 (0.49–3.41)	0.594
24	6.17 (2.17–17.55)	0.001	44	2.68 (0.97–7.40)	0.057
25	2.98 (0.98–9.04)	0.055	45	4.74 (1.56–14.36)	0.006
26	9.84 (3.40–28.48)	<0.001	46	9.52 (3.39–26.74)	<0.001
27	3.43 (1.10–10.70)	0.034	47	3.75 (1.40–10.06)	0.009
(**B**)					
11	2.72 (0.71–10.47)	0.147	31	1.00 Ref	-
12	1.34 (0.29–6.10)	0.706	32	1.74 (0.44–6.91)	0.432
13	1.34 (0.29–6.10)	0.706	33	1.74 (0.44–6.91)	0.432
14	2.37 (0.60–9.36)	0.219	34	1.98 (0.51–7.75)	0.328
15	2.72 (0.71–10.47)	0.146	35	2.35 (0.61–9.06)	0.215
16	3.42 (0.92–12.72)	0.066	36	3.07 (0.81–11.59)	0.098
17	3.42 (0.92–12.72)	0.066	37	4.50 (1.25–16.17)	0.021
21	3.07 (0.81–11.59)	0.098	41	1.74 (0.44–6.91)	0.432
22	2.37 (0.60–9.36)	0.219	42	1.74 (0.44–6.91)	0.432
23	2.13 (0.55–8.19)	0.272	43	1.74 (0.44–6.91)	0.432
24	3.07 (0.81–11.59)	0.098	44	2.35 (0.61–9.06)	0.215
25	2.13 (0.55–8.19)	0.272	45	2.72 (0.71–10.47)	0.147
26	4.14 (1.14–15.01)	0.031	46	4.14 (1.25–13.71)	0.020
27	3.42 (0.92–12.72)	0.066	47	3.78 (1.14–12.57)	0.030

Adjusted odds ratios (OR) and 95% confidence intervals were estimated using GEE logistic regression to account for clustering of teeth within participants. Models were adjusted for age category, sex, smoking status, ASA class, and medical history. Tooth 31 served as the reference category; third molars were excluded. *p*-value < 0.05 was considered statistically significant.

## Data Availability

The data presented in this study are available from the corresponding author upon reasonable request. Due to patient confidentiality and institutional regulations, the data are not publicly available.
